# Effect of high-potency statins on HbA1c in patients with or without diabetes mellitus

**DOI:** 10.1186/s40780-016-0040-0

**Published:** 2016-03-18

**Authors:** Nobuhiro Ooba, Shoutarou Tanaka, Yu Yasukawa, Nariyasu Yoshino, Hiroyuki Hayashi, Shinji Hidaka, Toshiichi Seki, Noriyasu Fukuoka

**Affiliations:** Department of Clinical Pharmacy, The Nihon University School of Pharmacy, 7-7-1 Narashinodai, Funabashi-shi, Chiba 274-8555 Japan; Department of Pharmacotherapy, The Nihon University School of Pharmacy, 7-7-1 Narashinodai, Funabashi-shi, Chiba 274-8555 Japan; Department of Pharmaceutical Regulatory Science, The Nihon University School of Pharmacy, 7-7-1 Narashinodai, Funabashi-shi, Chiba 274-8555 Japan; The Hitachinaka General Hospital, 20-1 Ishikawachou, Hitachinaka-shi, Ibaraki 312-0057 Japan

**Keywords:** High-potency statin, Hemoglobin A1c, New-user design

## Abstract

**Background:**

The increased risk of new-onset diabetes with statin use, including high-potency statins, is well known. However, the effects of high-potency statins on HbA1c are unclear. A retrospective cohort study was conducted to examine the effect of high-potency statins on HbA1c in patients with or without diabetes. The study enrolled new statin users identified via the electronic healthcare database of the general hospital in Japan.

**Methods:**

Following identification of all individuals (*n* = 4,672) who had been prescribed a lipid lowering drug at least once between January 1, 2010 and July 31, 2014, new statin users were selected (*n* = 1,136). Patients were excluded if they had been prescribed treatment with a statin within the preceding 6-month period. HbA1c levels before and during high-potency statin treatment were compared using the dependent t-test. In addition, the hazard ratio for the incidence of diabetes with high-potency statin treatment was estimated, using low-potency statins as a reference.

**Results:**

In patients with diabetes (*n* = 153), mean HbA1c (%) levels significantly increased by 0.4 % after high-potency statin use (7.57 ± 1.58; *p* = 0.0002) compared to baseline (7.18 ± 1.37). Similarly, HbA1c (%) levels significantly increased from 5.78 ± 0.38 to 5.92 ± 0.45 (*p* < 0.0001) after high-potency statin use in patients without diabetes (*n* = 165). Furthermore, a trend toward an increase in HbA1c was found for all of the high-potency statins irrespective of a history of diabetes.

**Conclusions:**

The use of high-potency statins may increase HbA1c levels in patients with or without diabetes.

## Background

Statins have been widely used for the prevention of cardiovascular disease [1, 2]. In Japan, the estimated number of patients with hyperlipidemia was approximately 1.9 million in 2011 [3]. It is essential to manage hyperlipidemia with statins to prevent the incidence of cardiovascular disease. However, statins are associated with major adverse effects including liver enzyme abnormalities and muscle toxicity, including rhabdomyolysis [4]. A meta-analysis by Sattar et al. [5] found that statin use was also associated with an increased risk of new-onset diabetes (overall odds ratio [OR] = 1.09; 95 % confidence interval [CI]: 1.02–1.17), and that the OR varied according to the potency of the statin. For high-potency statins (atorvastatin and rosuvastatin), the OR was 1.14–1.18, and for low-potency statins (simvastatin, pravastatin, and lovastatin), the OR was 0.98–1.11. A population-based retrospective cohort study in Canada that used administrative healthcare databases indicated that the risk of diabetes with high-potency statins was higher than with low-potency statins (hazard ratio [HR] = 1.22; 95%CI: 1.15–1.29) [6]. Therefore, the risk of new-onset diabetes may be different between low- and high-potency statins.

A recent meta-analysis found that statin treatment was associated with a modest increase in hemoglobin A1c (HbA1c, or glycated hemoglobin) in patients with diabetes [7]. Atorvastatin, a high-potency statin, had a particularly marked effect on HbA1c. A randomized controlled study of patients with type 2 diabetes conducted in Taiwan found that HbA1c was significantly increased after 3 months in patients receiving atorvastatin (6.5 % versus 6.6 %), while levels remained unchanged in those receiving pitavastatin (6.5 % versus 6.5 %), which has a similar potency [8]. On the other hand, in a randomized study of non-diabetic patients, neither of the high-potency statins investigated (atorvastatin and rosuvastatin) had a significant effect on HbA1c [9].

The effects on HbA1c might be different between different high-potency statins, and between patients with and without diabetes. Therefore, a retrospective cohort study was conducted to examine the effects of high-potency statins on HbA1c levels in patients with or without diabetes.

## Methods

### Study design and population

The data for this retrospective cohort study were obtained from the Hitachinaka General Hospital in Japan, which has approximately 300 beds. The data collected for each patient included study ID number; age and sex; ICD-10 (10th revision of the international classification of diseases) diagnosis codes; laboratory test results, including HbA1c (%); ordering laboratory tests for lipids; and the generic name, date, and number of days of supply of prescribed/dispensed drugs. A study ID number was created for each patient by the researcher within the study hospital.

**Fig. 1:**
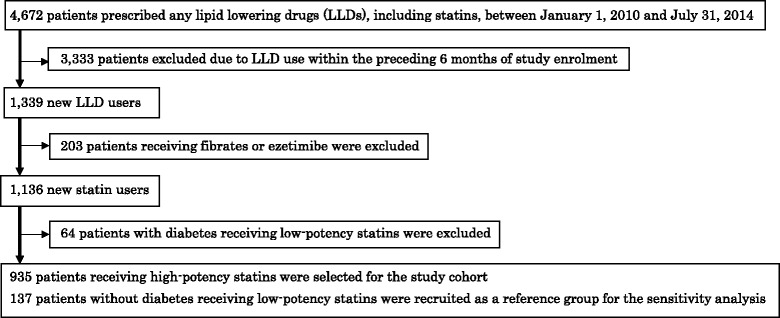
Patient selection flow

Figure 1 shows the patient selection flow. All patients with hyperlipidemia who had started treatment with a statin (after 6 months of non-use) in the study hospital were identified. First, patients who had been prescribed a lipid lowering drug at least once between January 1, 2010 and July 31, 2014 were identified by using the hospital information system, which included electronic prescription and medical records (diagnoses and the items or results of laboratory tests). Second, to identify the new statin users, patients were excluded if they had been prescribed the same or another statin in the preceding 6-month period. Pravastatin, simvastatin, and fluvastatin were defined as low-potency statins, and atorvastatin, pitavastatin, and rosuvastatin were defined as high-potency statins [6, 10]. Although data on the potency of pitavastatin is lacking, we considered pitavastatin as a high-potency statin because the reported effect of pitavastatin is similar to atorvastatin [11]. In addition, patients with diabetes receiving low-potency statins were excluded, as were patients receiving fibrates or ezetimibe. Finally, patients receiving high-potency statins were included in the study cohort for comparison of laboratory test results before and during statin treatment.

### Effects of high-potency statins on HbA1c

As this was an observational study using retrospective data, the doctors were not required to test for glycemic control prior to treatment with statins or at specific time points during treatment. The baseline HbA1c level was defined as the most recent measurement taken prior to initiation of low- or high-potency statin treatment. For each patient, the baseline value was compared with the HbA1c measurement taken after initiation of statin treatment; the time point for which varied in patients. In addition, the change in HbA1c was compared between patients with and without diabetes. To analyze HbA1c, we used the criteria set by the National Glycohemoglobin Standardization Program (NGSP). We also included the Japan Diabetes Society (JDS) criteria, as the NGSP HbA1c criteria are obtained by adding 0.4 % to the JDS HbA1c criteria [12].

For each patient, the start date was when statin treatment began. The study endpoint was defined as i) a new occurrence of diabetes (defined by an ICD-10 diagnosis code for diabetes or prescription of antidiabetic drugs including insulin), ii) discontinuation of statin treatment, iii) switching to another statin, or iv) July 31, 2014, whichever came first. Continuous statin use was defined using a grace period of up to 15 days between prescription dates.

### Ethics

The study protocol was approved by the ethics review committee of the Hitachinaka General Hospital (Ibaraki, Japan), and the Nihon University School of Pharmacy (No. 14–013). The waiver of informed consent from individual patients was approved by the ethics committee. Anonymized data with serial study ID numbers created by the study hospital were used throughout the study.

### Statistical analysis

The distribution of baseline characteristics during the preceding 6 months for new users of high-potency statins were described according to the history of diabetes. The definition of diabetes was based on the record of the ICD-10 code for diabetes or the prescription of antidiabetic drugs including insulin. A proportion including some covariates that may affect the incidence of diabetes, are shown in Table 1. The covariates included age, sex, mean follow-up (days), laboratory tests for HbA1c and lipids, use of concomitant drugs (systemic corticosteroids; thiazide diuretics; adrenergic beta-antagonists; antihypertensives including calcium-channel blockers [CCBs], angiotensin converting enzyme inhibitors [ACEIs] or angiotensin-receptor blockers [ARBs]; and antipsychotics), comorbidities (myocardial infarction, chronic heart failure, renal disease, liver disease, pulmonary disease, and hypertension), and Charlson Comorbidity Index [13]. To classify comorbidities [13], we used the following ICD-10 codes; diabetes (E10, E11, E13 and E14), hypertension (I10 and I15), myocardial infarction (I21, I22 and I23), chronic heart failure (I11), renal disease (I12, I13, N00 to N05, N11, N14, N17 to N19 and Q61), liver disease (B15, B16, B18, I85 and K70 to K76), and pulmonary disease (J40 to J47, J60 to J67, J70, J84, J96 and J98). The chi-square test was used to evaluate the differences between the high-potency statins. HbA1c levels before and during statin treatment were compared using the dependent t-test.

**Table 1:** Baseline characteristics for patients receiving high-potency statins

	Patients with diabetes (*n* = 276)				Patients without diabetes (*n* = 659)			
	Atorvastatin	Pitavastatin	Rosuvastatin	*P*-value	Atorvastatin	Pitavastatin	Rosuvastatin	*p*-value
Number of patients	87	70	119	-	221	151	287	-
Mean follow-up days	262	313	303	0.60	243	314	352	0.01
Male, %	75.9	65.7	62.2	0.11	52.0	53.0	57.5	0.43
Age, years, mean ± SD	66.2 ± 11	65.8 ± 12	67.5 ± 12	0.56	68.1 ± 11	67.4 ± 11	66.0 ± 12	0.12
Disease, %								
Diabetes	96.6	97.1	97.4	0.93	0	0	0	-
Hypertension	72.4	57.1	70.6	0.09	48.4	58.3	55.0	0.30
Myocardial infarction	12.6	11.4	18.5	0.33	17.6	17.2	19.5	0.80
Chronic heart failure	18.4	14.3	21.0	0.52	16.7	17.2	22.0	0.27
Renal disease	12.6	5.7	16.0	0.12	11.7	17.9	10.8	0.09
Liver disease	8.0	8.6	11.8	0.63	9.5	7.3	13.6	0.10
Pulmonary disease	13.8	14.3	12.6	0.94	14.9	13.2	19.1	0.22
CCI, mean	1.3	1.4	1.3	0.50	1.2	1.3	1.3	0.88
Concomitant medications, %								
Antidiabetics (insulin)	86.2 (10.3)	81.4 (10.0)	80.7 (8.6)	0.56	0	0	0	-
ACEIs or ARBs	47.1	47.1	47.1	0.99	35.3	40.4	33.8	0.39
Beta-blockers	25.3	10.0	31.9	0.003	21.2	26.5	22.0	0.45
Thiazides	4.6	8.6	6.8	0.60	4.5	1.3	5.9	0.08
CCBs	39.1	28.6	41.1	0.21	43.0	34.4	31.7	0.03
Antipsychotics	0	0	4.2	0.03	3.2	3.3	1.7	0.49
Laboratory tests								
HbA1c, %, mean (n)	6.9 (74)	7.3 (58)	7.0 (97)	0.14	5.7 (115)	5.8 (72)	5.8 (153)	0.67
Ordering Llipids, %	92.0	94.3	93.3	0.85	86.9	83.4	86.8	0.58

*Abbreviations*: *ACEIs* angiotensin converting enzyme inhibitors, *ARBs* angiotensin-receptor blockers, *CCBs* calcium-channel blockers, *CCI* Charlson Comorbidity Index, *HbA1c* hemoglobin A1c, *SD* standard deviation

For the sensitivity analysis, we restricted the population to patients without diabetes, and calculated the hazard ratio (HR) for new-onset diabetes with low-potency statins and high-potency statins using the Cox proportional hazards regression model. Unadjusted and multivariate-adjusted HRs were estimated with 95 % CIs for new-onset diabetes with high-potency statin treatment. A *p*-value <0.05 was considered statistically significant. All analyses were conducted using SAS, version 9.4 (SAS Institute, Cary, NC, USA).

## Results

### Baseline characteristics

In total, 1,136 new statin users were identified. Of these, 935 (82.3 %) were receiving a high-potency statin. Of the patients receiving high-potency statins, 276 (29.5 %) had a medical history of diabetes. Table 1 shows the baseline characteristics for the patients receiving high-potency statins by history of diabetes. Mean follow-up days of patients without diabetes were different between statin groups. The mean age was similar between patients with and without diabetes, while a larger proportion of patients with diabetes were male compared to patients without diabetes. The use of concomitant beta-blockers or antipsychotic drugs in patients with diabetes, and use of CCBs in patients without diabetes were significantly different between the high-potency statins. However, no other significant between-group differences were apparent.

### Changes in HbA1c in new users of high-potency statins

Table 2 shows the change in HbA1c with low-potency or high-potency statin treatment. With low-potency statin use as the control, 34 % (*n* = 22/64) of the patients with diabetes had HbA1c measurements for both before and during statin treatment while only 7 % (*n* = 10/137) of the patients without diabetes had both measurements available. With high-potency statin use, 55 % (*n* = 153/276) of the patients with diabetes had HbA1c measurements for both before and during statin treatment; 25 % (*n* = 165/659) of the patients without diabetes had both measurements available. Compared with the baseline value, the use of high-potency statins significantly increased HbA1c levels in patients both with and without diabetes. The effect on HbA1c levels was significantly higher in patients with diabetes compared to those without (*p* = 0.02). A similar trend was observed for each of the high-potency statin drugs. Although the use of low-potency statins tended to increase HbA1c levels in patients both with and without diabetes, the difference was not significant (*p* = 0.89).

**Table 2:** Change in HbA1c in new users of high-potency statins or low-potency statins as the control group

	High-potency statins	Atorvastatin	Pitavastatin	Rosuvastatin	Low-potency statins
With diabetes					
Number of patients	153	47	42	64	22
Mean period (days)^a^	209	166	189	254	231
HbA1c (%), mean ± SD					
Before	7.18 ± 1.37	7.03 ± 1.31	7.57 ± 1.64	7.03 ± 1.17	6.95 ± 1.92
After	7.57 ± 1.58	7.25 ± 1.21	8.11 ± 1.79	7.45 ± 1.61	7.25 ± 1.29
Difference	0.39 ± 1.27	0.21 ± 0.75	0.54 ± 1.61	0.42 ± 1.32	0.30 ± 0.94
*p*-value	0.0002	0.05	0.04	0.01	0.15
Without diabetes					
Number of patients	165	49	31	85	10
Mean period (days)^a^	239	212	310	229	236
HbA1c (%), mean ± SD					
Before	5.78 ± 0.38	5.76 ± 0.42	5.69 ± 0.24	5.81 ± 0.41	5.80 ± 0.47
After	5.92 ± 0.45	5.95 ± 0.45	5.90 ± 0.48	5.92 ± 0.45	6.07 ± 0.58
Difference	0.15 ± 0.31	0.19 ± 0.31	0.21 ± 0.46	0.10 ± 0.22	0.27 ± 0.18
*p*-value	<0.0001	0.0001	0.02	<0.0001	0.001
*p*-value^b^	0.02	−	−	−	0.89

*Abbreviations*: *SD* standard deviation

^a^Difference between statin start date and HbA1c measurement date

^b^Comparison of differences in HbA1c between diabetes and non-diabetes

### New-onset diabetes risk with high-potency statins

Table 3 shows the number of cases of new-onset diabetes and the unadjusted/adjusted HRs for diabetes associated with high-potency statins, using low-potency statins as a reference. The number of new cases of diabetes was small. Of the 11 cases of diabetes, 8 were identified by concomitant antidiabetic drug use in addition to an ICD-10 diagnostic code. The unadjusted/adjusted HRs for diabetes with high-potency statin treatment were 1.3 and 1.4, respectively; however, the 95 % CIs were wide.

**Table 3:** Hazard ratios for new-onset diabetes^a^ in patients receiving high-potency statins

	Number of patients	Number of cases	Crude HR (95 % CI)	Multiple adjusted^b^ HR (95 % CI)
Low-potency statins	137	1	Reference	Reference
High-potency statins	659	10	1.34 (0.17–10.50)	1.43 (0.18–11.74)

*Abbreviations*: *HR* hazard ratio, *CI* confidence interval

^a^Defined as diagnosis of diabetes or antidiabetic medication use

^b^Adjusted by age, sex, renal disease, and use of calcium-channel blockers

## Discussion

In this retrospective cohort study, we examined whether the use of high-potency statins may have an effect on HbA1c levels. The use of high-potency statins increased HbA1c levels irrespective of a history of diabetes. However, the degree of the effect of high-potency statins on HbA1c was significantly higher in patients with diabetes compared to those without diabetes. For the risk of new-onset diabetes, the adjusted HR for high-potency statins was 1.4, when low-potency statins were used as a reference; however, the 95 % CI was wide.

The risk of diabetes may differ between the low and high-potency statins. A meta-analysis [5] suggested that the risk of new-onset diabetes with high-potency statins (atorvastatin or rosuvastatin) may be modestly higher than with low-potency statins (pravastatin, simvastatin, or lovastatin). Furthermore, in an observational study using administrative healthcare databases, the risk of new-onset diabetes was found to be higher with high-potency statins than with low-potency statins [6]. Similar to those of previous studies [5, 6], our findings suggest that the risk of diabetes with high-potency statin treatment may be higher, although the 95 % CI was wide and the number of the cases of new-onset diabetes was small.

In our study, claims data and laboratory test results, including those for HbA1c, were available. A previous observational study [6] using claims database did not include laboratory test results. However, to examine the risk of diabetes with high-potency statin use, it is essential to compare HbA1c values before and after statin use.

We observed an increase in HbA1c with high-potency statins irrespective of a history of diabetes. Although the effect of pitavastatin, a high-potency statin, on glucose metabolism in patients with diabetes is under debate, the other two high-potency statins investigated here (atorvastatin and rosuvastatin) may also have an effect on HbA1c. The results of a meta-analysis on statins and glycemic control [7] and the findings of several other studies in patients with diabetes [14–16] suggest that statin treatment is associated with a modest increase in HbA1c. Our findings for patients with diabetes are consistent with these studies, with the exception of pitavastatin. On the other hand, the effect of high-potency statins on HbA1c in patients without diabetes may still be unclear, despite increased HbA1c levels observed in our study. A randomized trial of two high-potency statins (atorvastatin and rosuvastatin) in patients without diabetes reported that HbA1c levels were similar to baseline after 3 months of treatment [9]. In contrast, in another randomized trial of rosuvastatin, HbA1c levels increased from 5.7 to 5.9 % in patients without diabetes [17]. However, these randomized trials were not designed to evaluate the incidence of diabetes and the patient selection criteria were different than those in our study.

Our study has some limitations. First, the proportion of patients with laboratory test results, such as HbA1c, for both baseline and during statin treatment was low as this observational study was conducted in a real-world setting. The most common reason to order a laboratory test is to follow up on an abnormal finding or to monitor therapy [18]. As our study patients had hyperlipidemia, the proportion of laboratory tests performed to assess lipids at baseline was high (83 %). Around 80 % of the patients with diabetes had HbA1c measurements at baseline, but the proportion of patients without diabetes with HbA1c baseline measurements was approximately 50 %. In clinical practice, physicians might not monitor the effect of high-potency statins on glycemic control. Second, the diagnosis code for diabetes was not validated in our database, which may explain the uncertainty of the HR for diabetes with high-potency statin treatment. Although the number of new diabetes cases was small, 73 % of cases were recognized through concomitant antidiabetic drug use. Third, our study population, which was composed of new high-potency statin users, may lack generalizability as we used the data from a single district general hospital. However, all Japanese citizens are provided with care and treatment at any time and at the place of their choice, regardless of the type of facility, by universal health insurance coverage [19]. After we had identified all of the patients who had been prescribed lipid lowering drugs during the study period, we selected the new users. Therefore, there may be no difference between hospitals. Finally, the estimates for the risk of new-onset diabetes with high-potency statins were underpowered. Further studies are needed to examine the association between high-potency statins and diabetes using larger cohorts.

## Conclusions

The use of high-potency statins may increase HbA1c levels in patients irrespective of the presence of diabetes; however, the effect may be slightly more prominent in patients with diabetes. Treatment of hyperlipidemia with statins is important in the prevention of cardiovascular disease. However, our findings suggest that glycemic control should be closely monitored throughout treatment.
